# Development of high-efficiency superparamagnetic drug delivery system with MPI imaging capability

**DOI:** 10.3389/fbioe.2024.1382085

**Published:** 2024-03-20

**Authors:** Shi Bai, Xiao-dan Zhang, Yu-qi Zou, Yu-xi Lin, Zhi-yao Liu, Ke-wen Li, Ping Huang, Takashi Yoshida, Yi-li Liu, Ming-shan Li, Wei Zhang, Xiao-ju Wang, Min Zhang, Cheng Du

**Affiliations:** ^1^ Department of Information Engineering, Shenyang University of Technology, Shenyang, China; ^2^ Department of Electrical Engineering, Kyushu University, Fukuoka, Japan; ^3^ Department of Urology, Fourth Affiliated Hospital of China Medical University, Shenyang, China; ^4^ Department of Oncology, General Hospital of Northern Theater Command, Shenyang, China; ^5^ Department of Foreign Languages, Liaoning Vocational and Technical College of Economics, Shenyang, China; ^6^ First Affiliated Hospital, China Medical University, Shenyang, China

**Keywords:** drug delivery, magnetic drug targeting, magnetic particle imaging, superparamagnetic iron oxide nanoparticles, tumor therapy

## Abstract

In this study, a high-efficiency superparamagnetic drug delivery system was developed for preclinical treatment of bladder cancer in small animals. Two types of nanoparticles with magnetic particle imaging (MPI) capability, i.e., single- and multi-core superparamagnetic iron oxide nanoparticles (SPIONs), were selected and coupled with bladder anti-tumor drugs by a covalent coupling scheme. Owing to the minimal particle size, magnetic field strengths of 270 mT with a gradient of 3.2 T/m and 260 mT with a gradient of 3.7 T/m were found to be necessary to reach an average velocity of 2 mm/s for single- and multi-core SPIONs, respectively. To achieve this, a method of constructing an *in vitro* magnetic field for drug delivery was developed based on hollow multi-coils arranged coaxially in close rows, and magnetic field simulation was used to study the laws of the influence of the coil structure and parameters on the magnetic field. Using this method, a magnetic drug delivery system of single-core SPIONs was developed for rabbit bladder therapy. The delivery system consisted of three coaxially and equidistantly arranged coils with an inner diameter of Φ50 mm, radial height of 85 mm, and width of 15 mm that were positioned in close proximity to each other. CCK8 experimental results showed that the three types of drug-coupled SPION killed tumor cells effectively. By adjusting the axial and radial positions of the rabbit bladder within the inner hole of the delivery coil structure, the magnetic drugs injected could undergo two-dimensional delivery motions and were delivered and aggregated to the specified target location within 12 s, with an aggregation range of about 5 mm × 5 mm. In addition, the SPION distribution before and after delivery was imaged using a home-made open-bore MPI system that could realistically reflect the physical state. This study contributes to the development of local, rapid, and precise drug delivery and the visualization of this process during cancer therapy, and further research on MPI/delivery synchronization technology is planned for the future.

## 1 Introduction

Magnetic drug delivery has become a focus in the field of nanomagnetism in recent years (Hola et al., 2015; Patra et al., 2018; Aghebati-Maleki et al., 2020; Thakuria et al., 2021). Magnetic drugs, are also known as drug-coupled magnetic nanoparticles (MNPs) (Üzek et al., 2019; Hannachi et al., 2018; Y; Elbialy et al., 2019). Magnetic targeting involves inducing the magnetic drugs, *in vitro* under the action of a magnetic field, to move and accumulate at a lesion site (Vangijzegem et al., 2019; Li et al., 2021). This technique can significantly increase local drug concentration and achieve true local targeted therapy. Therefore, it has tremendous potential advantages in biomedical research (Dafeh et al., 2019; Elbialy et al., 2019; Mirza and Karim, 2021).

Many researchers have shown that magnetic drug delivery can be used for the treatment of various major diseases, including atherosclerosis (Haverkort et al., 2009), intramedullary spinal cord tumors (Kheirkhah et al., 2018), and metastatic breast cancer (Al Faraj et al., 2016). Sadeghzadeh et al. (2017) surface-modified superparamagnetic iron oxide nanoparticles (SPIONs) with copolymers and encapsulated curcumin to form drug-modified SPIONs. Simultaneously, the superparamagnetic properties of Fe_3_O_4_ nanoparticles were utilized to deliver curcumin towards lung tumor cells with the assistance of an external magnetic field. Alexiou et al. (2006) conducted magnetic drug delivery experiments in rabbits injected with tumors. Electron microscopy showed that a large number of MNPs accumulated in the tumor site with the assistance of a strong magnetic field gradient.

However, the main problem in magnetic drug delivery is a lack of design methods for targeting magnetic field strength and gradient. The enrichment of magnetic drugs may also cause side effects such as local thrombosis or vascular blockage. Moreover, the SPIONs used in the aforementioned study were too large (0.5–5.0 μm) (Rudge et al., 2000; Mishima et al., 2006) and were not suitable for dynamic tracking of magnetic drugs. These problems greatly limit the practical applications of this technology.

In the present study, efficient magnetic targeting conditions were theoretically analyzed and experimentally tested, and high-speed delivery of 30-nm single-core SPIONs was achieved by designing a coil structure in the drug delivery system. During the magnetic targeting process, magnetic particle imaging (MPI) technology was used to image the aggregation state of the magnetic drugs, providing an excellent empirical basis for the use of MPI to monitor the movement and aggregation state of magnetic drugs.

## 2 Materials and methods

### 2.1 Single- and multi-core SPIONs

In this study, two types of SPION, Resovist (Fujifilm, Japan) and Nanoeast (Nanjing Biotechnology Co., Ltd., China), were selected for magnetic drug delivery. The former is a commercial magnetic resonance imaging (MRI) T2 tracer that consists of an agglomerate of 5–7-nm γ-Fe_2_O_3_ elementary particles covered with carboxylated dextran, with an effective magnetic core size of about 25 nm (Yoshida et al., 2013; Assa et al., 2017). The latter comprises a single 30-nm Fe_3_O_4_ core with the same carboxylated dextran shell (Bai et al., 2023).

Surface modification of SPIONs is important for several reasons: (i) to provide the surface with functional groups required for stable drug coupling (Chen et al., 2011); (ii) to ensure the particles are biocompatible and reach the tumor area without being recognized by the immune system when used *in vivo* (Kim J. E. et al., 2012); (iii) to maintain good dispersibility without aggregation and precipitation under normal conditions; and (iv) to reduce the toxic side effects of magnetic cores, while ensuring that they are not further oxidized and maintaining magnetic stability (Assa et al., 2017).

**FIGURE 1:**
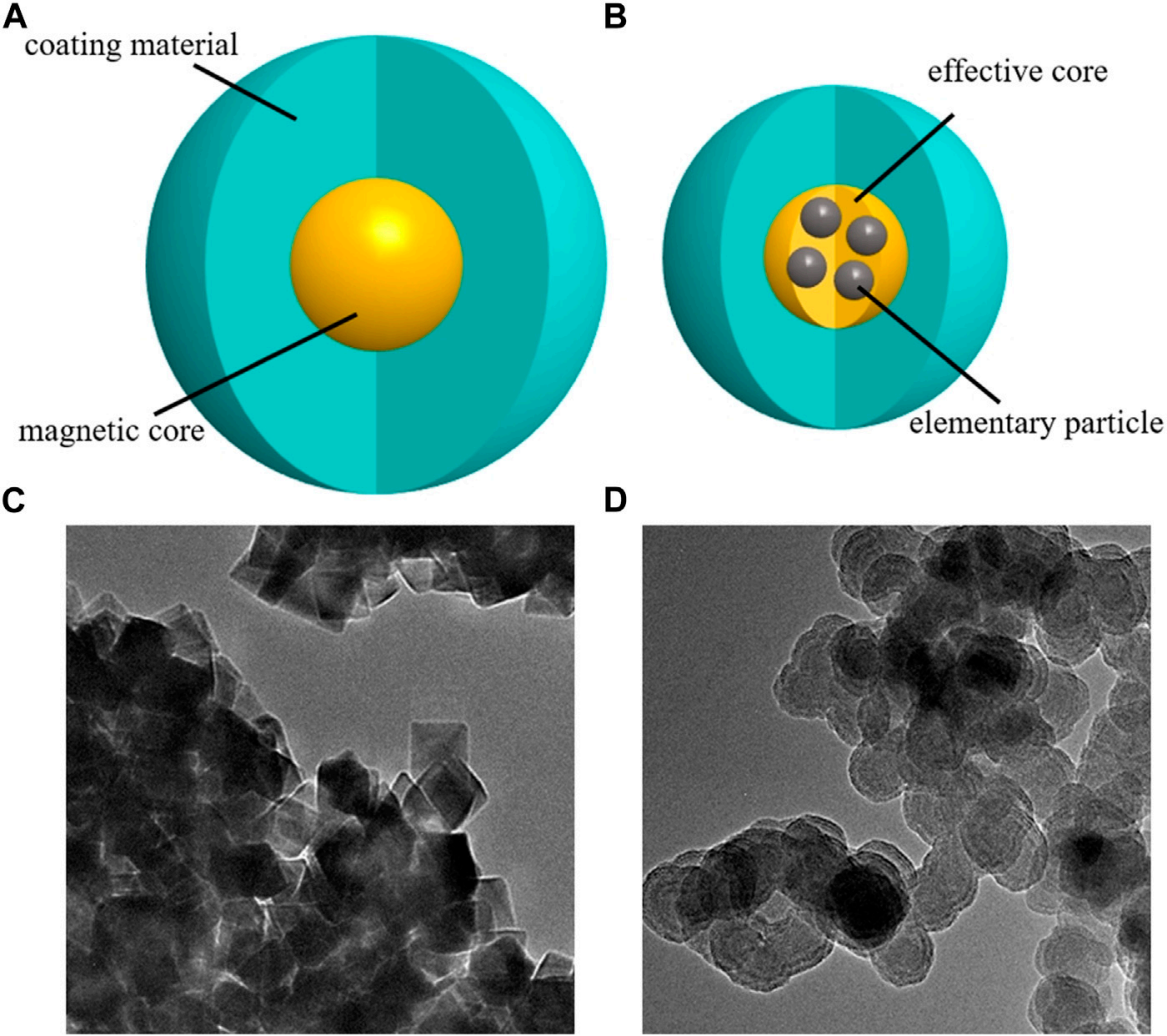
Schematics and images of superparamagnetic iron oxide nanoparticles. **(A)** single-core structure; **(B)** multi-core structure. **(C)** Transmission electron microscopy image of Nanoeast, with a magnetic core of 30 nm Fe_3_O_4_, covered with carboxylated dextran; the hydrodynamic diameter is 80 nm. **(D)** Transmission electron microscopy image of Resovist; the magnetic core consists of an agglomerate of 5–7 nm γ-Fe_2_O_3_ elementary particles, covered with carboxylated dextran, the effective magnetic core size is about 25 nm, and the hydrodynamic diameter is 58 nm.

Resovist and Nanoeast have hydrodynamic diameters of 58 nm and 80 nm, respectively, as measured by dynamic light scattering (Enpuku et al., 2017). Both SPIONs have a particle size distribution that is uniform and well dispersed. A schematic of the structure and transmission electron microscopy results are presented in Figure 1. In addition, both SPIONs exhibit good superparamagnetism (Leslie-Pelecky and Rieke, 1996; Krishnan et al., 2006; Lai et al., 2008; Qiao et al., 2009; He et al., 2013; Kim M. et al., 2019). When the external magnetic field is removed, the magnetism of the SPIONs also disappears, which effectively avoids the blocking phenomenon. Furthermore, both SPIONs support an MPI imaging function (Molwitz et al., 2019; Billings et al., 2021), and the carboxyl groups on their surface support drug modification (Dramou et al., 2013; Hola et al., 2015). Moreover, owing to different magnetic core sizes and magnetic anisotropy (Coffey et al., 1993; Dormann et al., 1997; Vasir and Labhasetwar, 2007; Chu et al., 2015; Tan et al., 2018), they behave differently in magnetic drug targeting systems; this will be described in detail in the following sections.

### 2.2 MPI system

The self-developed open-bore preclinical MPI device (OpenMagic, Jiayu Technology, China) was used to image the distribution of SPIONs before and after magnetic targeting. The maximum field of view was about 100 mm × 100 mm with a gradient field of 4 T/m, corresponding to a spatial resolution of about 1 mm after reconstruction. A 4 T/m gradient field was generated by a direct current of 10 A, with an excitation signal at 8,461 Hz consisting of a sinusoidal wave of 20 A, and a detection signal as the third harmonic.

To validate the feasibility of magnetic drug imaging using MPI *in vivo*, an 8-week-old female rat was anesthetized by intraperitoneal injection of pentobarbital sodium (40 mg/kg weight), and a drainage tube was inserted into its bladder. Subsequently, epirubicin (epi)- modified Nanoeast 30 nm SPIONs (20 μg Fe) were injected into the bladder through the drainage 1tube and left for 2 min before the bladder area was imaged using an open-bore MPI device.

### 2.3 Drug coupling

Three chemotherapeutic agents, epi, mitomycin (mmc), and hydroxycamptothecin (hcpt), which are often used in bladder cancer treatment, were connected to the SPIONs’ surface. The functional groups on the surface of both SPION types was COOH; thus, epi and mmc were connected to the SPIONs by a CO-NH reaction, and hcpt was connected by dehydration condensation (Hola et al., 2015).

To evaluate the drug-coupling effect, epi and Nanoeast were chosen for drug-coupling rate testing. A combination of magnetic drugs was formed by adding 100 μg, 50 μg, 20 μg, 10 μg, 5 μg, or 1 μg of epi to every 100 μg of Nanoeast SPIONs. An enzyme marker was used to measure the absorbance, and the drug-coupling rate was then determined based on the absorbance change.

### 2.4 Magnetic drug evaluation

The bioavailability of magnetic drugs was evaluated in a bladder cancer cell-killing experiment, and it was demonstrated that the drugs were released over time. T24 bladder cancer cells were pre-cultured in 96-well plates, then injected with 10 μL nothing (control group: no injection), uncoupled Nanoeast (nc1), uncoupled Resovist (nc2), and 24 h half-maximal inhibitory concentrations of the anti-tumor drugs (epi, mmc, and hcpt) and magnetic drugs (Nanoeast coupled to the anti-tumor drugs to form magnetic drugs n-epi, n-mmc, and n-hcpt) and treated for 24 h. The experiment was repeated six times for each group.

The cytotoxicity and safety of SPIONs with respect to cells were assessed through microscopic observations and a CCK-8 proliferation assay. CCK-8 reaction reagent (10 μL) and culture medium (90 μL) were added to each 96-well plate. The reaction proceeded for 2 h under dark conditions at 37 °C, after which the absorbance was measured at a single wavelength of 450 nm, and the cell survival rate was calculated.

### 2.5 Calculation of magnetic force under external magnetic field

There are two main types of force acting on suspended SPIONs under an external delivery magnetic field, i.e., magnetic force $F_{m}$ and fluid viscous resistance $F_{s}$ . The resultant force of $F_{m}$ and $F_{s}$ produces the velocity and acceleration of the SPIONs. As the speed of the SPIONs increases, $F_{s}$ increases gradually. When the magnetic force is eventually equal to the fluid viscous resistance, the SPIONs move at a constant speed.

$$F_{m}+F_{s}=m_{p}∙\frac{dv_{p}}{dt}$$

where $m_{p}$ is the mass of the SPIONs, and $v_{p}$ is the kinematic velocity, with

$$F_{m}=V_{p}\left(\chi_{p}-\chi_{f}\right)H∙\nabla B$$

and

$$F_{s}=3\pi \eta d_{H}v_{p}$$

where $V_{p}$ is the volume of the nanoparticles; $\chi_{p}$ and $\chi_{f}$ are the magnetic susceptibility of the nanoparticles and fluids, respectively; H is the applied magnetic field strength; ∇B is the magnetic field gradient; η is the fluid kinematic viscosity; and $d_{H}$ is the hydrodynamic diameter of the SPIONs.

The nanoparticles are magnetized and then deflected in a uniform magnetic field but do not move ( ∇B = 0, $F_{m}$ = 0); they only move in a gradient magnetic field ( ∇B ≠ 0, $F_{m}$ ≠ 0). The nanoparticles can be extracted from the fluid and accumulate and are retained at the target site owing to the difference between the magnetic susceptibility of the nanoparticle and that of the fluid (the magnetic susceptibility of the fluid in this system is close to zero). As H increases, $F_{m}$ gradually increases. When H increases to a certain value, the magnetization ***M =*** $\chi_{p}\;H$ reaches saturation, and the magnetic force $F_{m}$ no longer changes with H but is related to the magnetic field gradient ∇B . Therefore, to obtain better therapeutic effects in magnetic targeted therapy, nanoparticles with large magnetic susceptibility should be selected as drug carriers, and the *in vitro* targeting magnetic field should have a large magnetic field gradient and a magnetic field strength that meets requirements.

The movement of SPIONs, both accelerated and uniform, is affected by the magnetic moment m , the magnetic field strength, and the gradient. For spherical SPIONs, m is given by

$$m=\frac{\pi M_{s}}{6}d_{c}^{3}$$

where $M_{s}$ is the saturation magnetization, and *d*_c_ is the diameter of the magnetic core. The larger the magnetic core size, the larger the value of m , and the greater the magnetic force required to deflect and move the particle. Anisotropic energy E needs to be overcome when m is deflected in the direction of the applied magnetic field, as follows:

$$E=\frac{\pi }{6}d_{c}^{3}K$$

where K is the anisotropy energy density; K = 5 kJ·m^−3^ and 13 kJ·m^−3^ for Resovist (multi-core) and Nanoeast SPIONs (single core), respectively (Enpuku et al., 2016). The magnetic field force is used to overcome E , which deflects m , causing the SPIONs to move. Nanoeast single core SPIONs have a larger magnetic core size in comparison with Resovist multi-core SPIONs, resulting in greater magnetic field force (or magnetic field strength) being required for their motion. The targeting efficiency of Nanoeast was found to be superior to that of Resovist at the same magnetic field strength.

In addition, in the static magnetic field used for targeting, the magnetic field force $F_{m0}$ , which overcomes the static friction force and makes the SPIONs start to move, is much larger than the magnetic field force $F_{m}$ , which overcomes the fluid viscous resistance and makes SPIONs keep moving. Therefore, in order for SPIONs at rest to start moving, a large magnetic field strength is required. Furthermore, SPIONs have a tendency to stick to cell surfaces once they have been injected into the body, which is likely to occur in practical clinical applications. It is therefore crucial to generate an initial force $F_{m0}$ that is greater than $F_{m}$ to overcome the maximum static friction and/or cell adhesion forces.

Thus, the movement of suspended SPIONs is defined as follows:

$$F_{m0}>F_{m}=M∙\nabla B\propto M,\nabla B$$

$$m\propto d_{c}^{3}\propto V_{p}$$

$$E\propto d_{c}^{3}$$

$$E\propto B_{\text{expect}}$$

where $B_{\text{expect}}$ represents the external magnetic field strength required for the SPIONs to overcome E . Specifically, for single-core and multi-core SPIONs, the anisotropy energy E that must be overcome to deflect a multi-core Resovist with an equivalent magnetic core diameter of 25 nm in the external magnetic field direction is 4.09 × 10^−20^ J. This is an order of magnitude smaller than the E = 1.84 × 10^−19^ J that must be overcome when a single-core Nanoeast with a magnetic core diameter of 30 nm is deflected in the direction of the external magnetic field. In addition, the $d_{c}$ and $d_{H}$ ( m and $F_{s}$ ) of multi-core Resovist are smaller than those of single-core Nanoeast. Thus, targeting motion can be performed at a relatively low speed with a lower external magnetic field strength using Resovist, whereas Nanoeast requires a stronger external magnetic field strength to achieve targeting motion at a higher speed. The magnetic field gradient is equally important in each case. The magnetic field strength initiates the motion of the SPIONs, whereas the magnetic field gradient determines their acceleration, which is a combination effect.

### 2.6 Magnetic targeting test

To simulate the operating environment of SPIONs and magnetic drugs (Nanoeast coupled mmc, n-mmc, and Resovist coupled mmc, r-mmc) *in vivo*, saline was used to dilute them according to a specific ratio. After dilution, the Nanoeast and Resovist concentrations were 3.11 mg/mL and 12.51 mg/mL, respectively. The diluted salt solutions of SPIONs and magnetic drug were then injected into Φ4 mm drainage tubes. Two kinds of SPIONs salt solution were placed in different magnetic field strengths and gradients for magnetic targeting experiments. Two kinds of magnetic drug salt solution were placed in different axial positions in the inner hole of the developed delivery coil structure to verify the delivery ability.

The delivery distance and time of SPIONs were recorded under different magnetic field parameters. To characterize magnetic drug delivery efficiency, the average motion speed of SPIONs was calculated as the distance divided by the time. Although individual SPIONs, or magnetic drugs, are nanoscale and invisible to the naked eye. However, when a large number of SPIONs or magnetic drugs are aggregated under the action of a magnetic field, agglomerates can be formed that are visible to the naked eye. Therefore, the measurement of their movement distance can be achieved through macroscopic observation.

## 3 Development of magnetic drug delivery system

To investigate the movement of SPIONs under an external magnetic field and determine the necessary parameters for magnetic drug delivery, such that the magnetic drug can quickly accumulate and remain in the tumor area under the action of the external magnetic field to increase the local drug concentration in the tumor area, a thorough study was conducted of the magnetic field parameters and coil structure design. The movements of the two types of SPION were tested under different magnetic field strengths and gradients. The alterations in their motion conditions, based on the magnetic field strength and gradient, were observed to ascertain the necessary *in vitro* magnetic field parameters for magnetic drug delivery. The coils’ structural form and parameters were simulated and tested to design an *in vitro* magnetic field structure compatible with magnetic drug delivery and ensure that the magnetic field strength, gradient, target size, and inner diameter met the necessary requirements.

### 3.1 Key parameters of delivery magnetic field

In this part of the study, the effects of the magnetic field strength and gradient on the delivery efficiency of different SPIONs were investigated to determine the key delivery magnetic field parameters. The delivery time and distance were measured for the two types of SPION under the same conditions of magnetic field strength and gradient, respectively. The delivery efficiency of the SPIONs was evaluated indirectly based on their average speed of movement to determine the optimal delivery magnetic field parameters.

**FIGURE 2:**
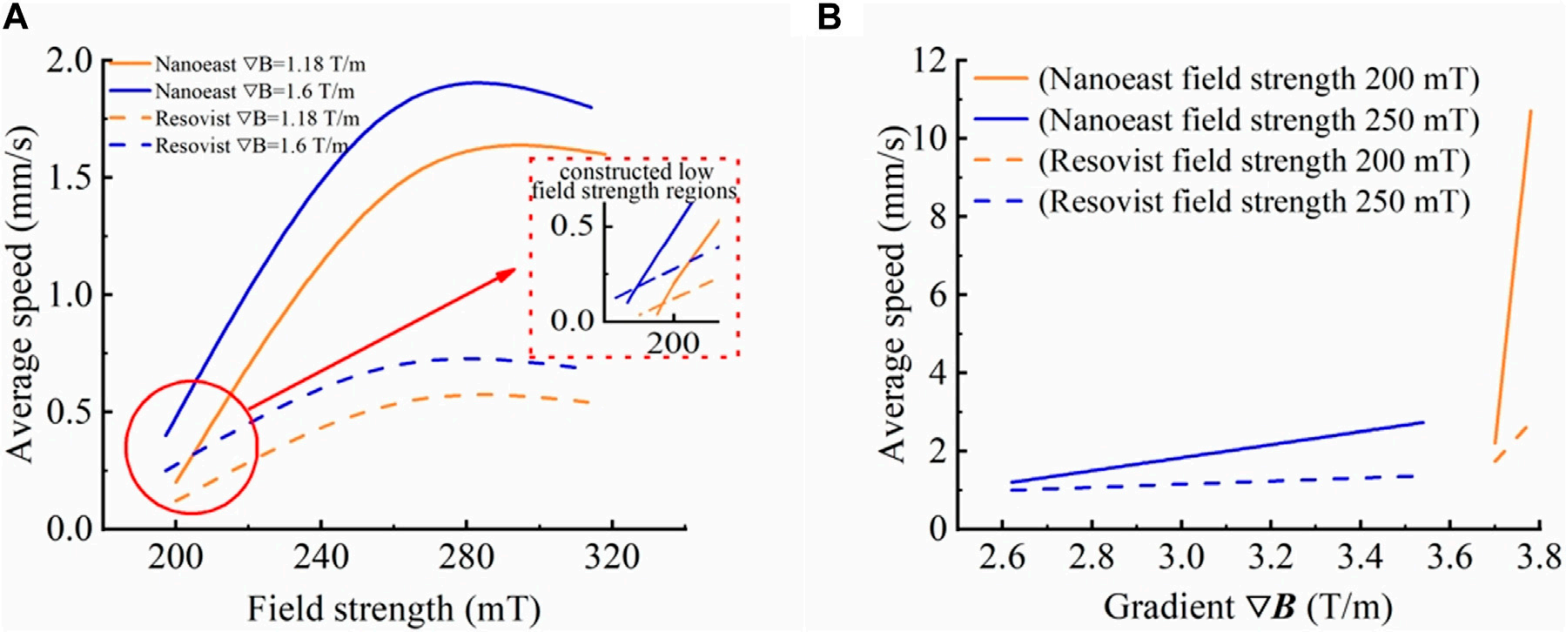
Movement velocities of Nanoeast and Resovist in saline solution with different magnetic field parameters. **(A)** Effects of magnetic field strength on the motion velocity of single-core Nanoeast and multi-core Resovist with the same magnetic field gradient. The magnetic field gradients were 1.18 T/m and 1.6 T/m, respectively. **(B)** Effects of magnetic field gradient on the motion velocity of single-core Nanoeast and multi-core Resovist with the same magnetic field strength. The magnetic field strengths were 200 mT and 250 mT, respectively.

Under the same gradient, as the magnetic field strength increased, the average movement velocity of both SPIONs showed a trend of increasing and then stabilizing. Notably, the velocity inflection points of single-core Nanoeast and multi-core Resovist were located at 270 mT and 260 mT, respectively, as shown in Figure 2A. Overall, as the field strength increased, the velocity alteration of Nanoeast was greater and faster, whereas that of Resovist tended to be comparatively slow. Under identical magnetic field conditions, Nanoeast displayed higher movement velocities than Resovist; the velocity difference between the two gradually increased with increasing magnetic field strength and eventually remained constant. On the basis of the test results, a low field strength region was constructed. When the field strength was below a certain value, the motion rate of multi-core Resovist was faster than that of single-core Nanoeast.

This was because when the magnetic force exceeds the anisotropic energy and maximum static friction force, the magnetic moment of the SPIONs is deflected, inducing targeted motion. The small magnetic core size of Resovist results in a reduction of anisotropic energy. As a result, it begins to move first when the applied magnetic field is small, making it move faster than Nanoeast initially. As the magnetic field strength increases, Nanoeast begins to move. Owing to its larger magnetic core size and single core structure, Nanoeast is subjected to a greater magnetic field force and accelerates faster for the same magnetic field parameter, and its speed soon becomes faster than that of Resovist. With further increase in magnetic field strength, the magnetic force increases, causing the SPIONs to move even more quickly and leading to an increase in the fluid viscous resistance $F_{s}$ . When the fluid viscous resistance balances the magnetic force, SPIONs undergo uniform motion. In addition, when the magnetic field strength is greater than a certain value, the SPIONs are in a state of saturated magnetization, and their magnetic field force does not change with the increase of magnetic field strength. Therefore, in summary, there exists an inflection point on the velocity curve, beyond which the velocity basically stays the same.

At a given magnetic field strength, the average motion velocity of both SPIONs tended to increase as the magnetic field gradient increased. A gradual increase was noted for small gradients, whereas for large gradients, the velocity increased rapidly, reaching an inflection point at about 3.6 T/m, as shown in Figure 2B. This was because in a gradient magnetic field, a region with a high gradient is close to the peak point of the magnetic field strength and possesses a strong magnetic field strength. Under the dual effect of magnetic field gradient and strength, the magnetic field force increases rapidly, and a small change in gradient causes a huge change in the velocity of SPIONs.

Furthermore, there was a crossover in SPION motion velocities under the two conditions of lower magnetic field strength and higher gradient, and higher magnetic field strength and lower gradient. For instance, Nanoeast exhibited an average motion rate of 2.5 mm/s under conditions of 250 mT with 3.4 T/m and of 200 mT with 3.7 T/m. As demonstrated by equation (2), the magnetic field force is dependent on both the strength and gradient of the magnetic field. The magnetic field strength comes into play before the nanoparticles reach saturation magnetization, whereas the magnetic field gradient is not limited. Thus, the delivery efficiency of SPIONs can be managed by adjusting these factors. Augmenting the magnetic field strength initiates movement of SPIONs in the stationary state and enhances their speed in the moving state. Increasing the magnetic field gradient enhances the magnetic field force, which in turn increases the motion speed of the SPIONs. However, as the magnetic field gradient is typically small (often less than 10 T/m), it may have little effect on the alteration in the magnetic field force. Consequently, the change in the SPIONs’ motion speed is relatively gradual. By contrast, a significant value greatly affects the magnetic force and causes the SPIONs’ motion speed to rapidly change. It should be noted that the magnetic force is affected by both the strength and gradient of the magnetic field, resulting in a combined effect. A single parameter adjustment, such as increasing strength or decreasing gradient, can bring about equivalent changes in the delivery magnetic field parameters, allowing SPIONs to move at the same speed. This discovery removes limitations on the structure and parameters of the delivery magnetic field, resulting in more diversified delivery magnetic field structures. This provides an opportunity to design more flexible delivery magnetic field structures and conveniently control the delivery magnetic field parameters.

The magnetic core size of Resovist multi-core SPIONs is smaller than that of Nanoeast single core SPIONs; therefore, the magnetic field force required to overcome the anisotropic energy to deflect the magnetic moment is lower. Owing to its weaker magnetic force, Resovist moves correspondingly slower. When choosing magnetic drug carriers for targeted therapy, it is recommended to prioritize multi-core, small-sized SPIONs such as Resovist for use with a weaker applied targeting magnetic field. For a stronger delivery magnetic field, single-core, larger-sized SPIONs such as Nanoeast may be more appropriate. It is essential to consider these factors when selecting the appropriate magnetic drug carrier for targeted therapy. The experimental results and analysis presented here reveal that magnetic drug targeting is subject to various factors, including the properties of the material itself and the strength and gradient of the applied magnetic field. When using Nanoeast and Resovist, magnetic drug targeting delivery time can be effectively reduced, and the average speed can exceed 2 mm/s when the magnetic field strength and gradient are at least 270 mT and 3.2 T/m, and 260 mT and 3.7 T/m, respectively. These indices provide a reference for the design of magnetic targeting systems.

### 3.2 Development of delivery system

Magnetic field parameters are crucial in magnetic targeting therapy. The delivery coils are a concrete realization of magnetic field parameters, and their structure and parameter design form the core of magnetic drug targeting therapy.

The theoretical analysis in Section 2.5 and the delivery magnetic field parameter experiment results presented in Section 3.1 make it evident that the magnetic strength and gradient have crucial roles in the motion of SPIONs, determining their ability to move and the speed at which they do so. The size of the area following SPIONs aggregation, which represents the effective range of action of chemotherapeutic agents, is determined by the size of the target area of the magnetic field. If the magnetic field target area is too large—that is, larger than the size of the tumor area—the chemotherapeutic agent will aggregate and be released in the normal tissues around the tumor, which could be extremely dangerous and damaging to the animal or human body. On the contrary, if the target area of the magnetic field is too small—that is, smaller than the size of the tumor area—this not only adds difficulty to the design of the delivery coil structure, but the delivery and therapeutic effects are also unsatisfactory. In addition, the inner hole of the delivery coil structure is used to place isolated organs or animal or human bodies (hereafter referred to as the object to be examined) in order to conduct *ex vivo*, *in vitro*, and *in vivo* experiments. If the inner hole of the coil structure is too small, it will not be able to accommodate the object to be examined, or it will not be easy to operate or observe, which will be detrimental to the experimental experience. If the inner hole of the coil structure is too large, it will be difficult to ensure the strength and gradient of the magnetic field. The number of ampere-turns needed to produce the same magnetic field will increase, causing the coil to heat up more and resulting in wasted resources, which is not favorable for practical applications. Moreover, the difference in radial magnetic field strength increases with changes in position. Therefore, the therapeutic effect is slightly different when the object to be examined is placed in different radial positions in the inner hole of the coil structure. The magnetic field parameters directly affect the movement and aggregation of magnetic drugs, which in turn influence the effects of magnetic targeting therapy. Therefore, the design of the structure and parameters of the delivery coil is key to ensuring the effectiveness of magnetic targeting therapy.

#### 3.2.1 Multi-coil delivery structure

##### 3.2.1.1 Structure of delivery coil

Traditional magnetic targeting therapy often involves placing permanent magnets outside the body (Widder et al., 1983; Gupta and Hung, 1990; Lübbe et al., 1996a) so that the magnetic drug accumulates at the tumor. However, this method is affected by the rapid attenuation of magnetic field strength with distance and can only be used to treat tumors on the body surface and in the superficial layers. At present, the application of permanent magnets *in vitro* is often optimized and improved by implanting tiny permanent magnets near the tumor (Babincová et al., 2000; Kubo et al., 2001; Asmatulu et al., 2005; Yellen et al., 2005). However, this method represents an invasive treatment and requires the implantation of permanent magnets into the body through surgery, which is risky.

**FIGURE 3:**
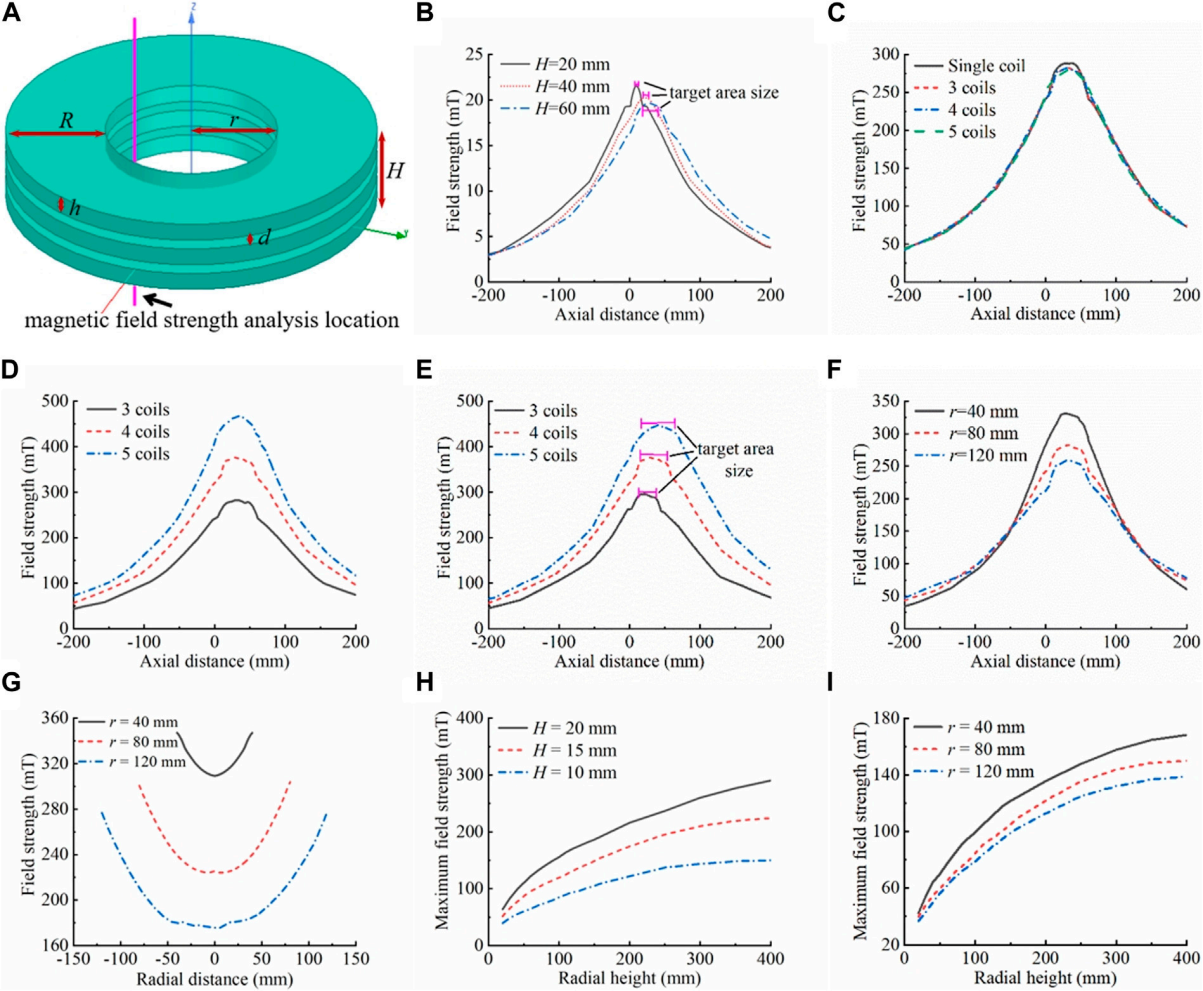
Delivery coil structure simulation analysis. **(A)** Schematic diagram of the delivery coil structure, which is made of hollow multi-layer narrow coils arranged coaxially in close proximity. The inner diameter of the coil is *r*, and the radial height is *R*, the width of a single coil is *h*, the spacing between neighboring coils is *d*, the overall width of the coil is *H*, the number of coils is *N*, and the number of ampere-turns is *AN*. The entire coil has the center of the bottom surface of the lowest coil as the origin. The magnetic field strength was analyzed in the inner hole, 10 mm from the inner wall of the coil, and 200 mm above and below the origin along the axial direction. **(B)** Effects of individual coil width on the magnetic field. Keeping *r* = 80 mm, *R* = 200 mm, *N* = 1, *AN* = 4,000 A·N unchanged, the effects of the coil width on the magnetic field (magnetic field strength, gradient, length of the uniform magnetic field, etc.) were analyzed, where *h* = *H* = 20/40/60 mm. **(C)** Equivalent replacement of a single coil. Keeping *r* = 80 mm, *R* = 200 mm, *d* = 5 mm, *H* = 65 mm, and total *AN* = 60,000 A·N constant, the difference in the magnetic field between a single coil (*N* = 1, *h* = 65 mm, *AN* = 60,000 A·N) and the equivalent replacement with multiple coils (*N* = 3, *h* = 18.33 mm, each coil *AN* = 20,000 A·N; *N* = 4, *h* = 12.5 mm, each coil *AN* = 15,000 A·N; *N* = 5, *h* = 9 mm, each coil *AN* = 12,000 A·N) arranged coaxially in close proximity was analyzed. **(D)** Effects of increasing the number of individual coil ampere-turns on the magnetic field. The *AN* of each coil in **(C)** was increased to 20,000 A·N, *N* = 3 was kept constant. At *N* = 4, the individual coil *AN* increased from 15,000 A·N to 20,000 A·N, an increase of 5,000 A·N, and the magnetic field strength increased by about 93 mT. At *N* = 5, the individual coil *AN* increased from 12,000 A·N to 20,000 A·N, an increase of 8,000 A·N, and the magnetic field strength increased by about 184 mT. The model can also be used to analyze the effects of the number of disassembled coils on the magnetic field (for the same number of individual coil ampere-turns and overall coil width). The greater the number of disassembled coils, the more ampere-turns per coil can be boosted. The greater the number of coils disassembled, the greater the increase in magnetic field strength and gradient, when boosted to the same number of ampere-turns (individual coils). **(E)** Effects of the overall width (number of coils) of the delivery coil structure on the magnetic field. Keeping *r* = 80 mm, *R* = 200 mm, *h* = 12.5 mm, *d* = 5 mm, and *AN* = 20,000 A·N unchanged, the effects of the overall width of the coils (the number of coils) on the magnetic field were analyzed, with *N* = 3/4/5. **(F)** Effects of coil inner diameter on the magnetic field. Keeping *R* = 200 mm, *d* = 5 mm, *H* = 65 mm, *h* = 18.33 mm, *N* = 3, and individual coil *AN* = 20,000 A·N unchanged, the effects of the inner diameter size on the magnetic field were analyzed, where *r* = 40/80/120 mm. **(G)** Effects of coil inner diameter on internal radial magnetic field differences. The analyzed position of the magnetic field strength is the diameter of the inner hole in the *x*-*y* plane at the axial center of the coil combination system, i.e., the diameter of the inner hole in the plane of *z* = 32.5 mm. With other parameters unchanged, the larger the size of the inner hole of the coil, the larger the total change value of the internal radial magnetic field strength (on the basis of **(F)**, *r* = 40 mm, maximum change in the internal radial magnetic field strength of 37.95 mT; *r* = 80 mm, maximum change in the internal radial magnetic field strength of 76.22 mT; *r* = 120 mm, maximum change in the internal radial magnetic field strength of 100.97 mT). **(H)** Effects of radial height of the coil on the magnetic field (comparing different coil widths (left) and inner diameters (right)). For different widths: *r* = 80 mm, *N* = 1, single-turn excitation line passes 10 A current. For different inner diameters: *H* = 10 mm, *N* = 1, single-turn excitation line passes 10 A current. As the radial height of the coil increases, the magnetic field strength in the inner hole of the coil increases rapidly and then increases slightly, or even remains almost unchanged. The magnetic field inflection point is generally located in the range of 200–300 mm.

In this study, to increase the general applicability of magnetic targeting therapy to effectively treat deep tissues, the object to be examined was placed in an excitation coil, and the uniform magnetic field in the axial direction of the excited coil, as well as the small magnetic field changes (compared with those of permanent magnets) in the radial direction, were used to effectively solve the problem described above. The structure of the delivery coil is shown in Figure 3A.

Furthermore, when the coil was subjected to the same number of ampere-turns, a decrease in coil height resulted in a more concentrated and stronger magnetic field, with a higher gradient. This shorter axial uniform magnetic field length allowed for a more compact and precise target area design. The Maxwell module of the Ansys Electronic Desktop simulation software was used for the simulation; the simulation results are shown in Figure 3B. Nonetheless, when the number of ampere-turns of a single coil reaches a certain level, the magnetic field strength and gradient generated by the coil may not be able to meet requirements owing to increased coil resistance, serious heat generation, and limited power supply. To tackle this phenomenon, individual coils could be replaced by multiple coils closely aligned coaxially, which would increase the number of ampere-turns of each coil and ultimately increase the strength and gradient of the overall magnetic field. The simulation results in Figure 3C show that when a single coil is divided into multiple coils, if the total height of the coils and the total number of ampere-turns are kept constant, the magnetic field strength is slightly reduced (by about 6 mT) compared with that of a single coil, which is negligible. These results thus demonstrate the feasibility of the method of coil disassembly. For disassembled multiple coils, the total number of ampere-turns of each coil can be increased by increasing the number of winding turns, the number of power supplies, and the supply current to improve the total magnetic field strength and gradient. Figure 3D shows the effectiveness of this approach in significantly enhancing magnetic field parameters. Simultaneously, disassembling more coils allows for greater capacity to increase the number of ampere-turns per coil. When upgrading to the same number of ampere-turns (single coil), disassembling more coils results in a greater increase in magnetic field strength and gradient. However, this also leads to an increase in the number of excitation power supplies and the cost. Therefore, when disassembling the coils and designing the number of coils, on the premise that the magnetic field meets the demand, the number of coils should be as small as possible in order to reduce the complexity of the system and save costs.

Based on the considerations above, the delivery coil structure was designed as a hollow multi-coil arranged coaxially in close proximity. This enhanced the flexibility of operation and application and expanded the potential applications of the magnetic targeting therapy system to different objects to be examined. Furthermore, there was a significant reduction in the height of the individual coils. The magnetic field strength and gradient could be effectively enhanced and flexibly adjusted by changing the number of excitation coils or increasing the number of turns of winding coil, the number of power supplies, and the supply current. This allows for precise targeted therapy for small tumors while further reducing the size of the target area.

##### 3.2.1.2 Width of delivery coil

The width of the coil has two aspects: one relating to the overall width of the multi-coil structure and the other relating to the width of the individual coils that make up the multi-coil structure. The simulation results in Figure 3E show that the greater the number of coils, i.e., the larger the overall width of the multi-coil structure, the larger the maximum values of the magnetic field strength and gradient; moreover, the length of the region of uniform magnetic field with the maximum field strength (the target area) is longer, and the size of the target area is smaller than the overall width of the multi-coils. In the axial direction of the multi-coil structure, the magnetic field strength followed an axisymmetric law of change characterized by a “rapidly increasing–basically unchanged–rapidly decreasing” pattern. The axis of symmetry was located in the axial center of the excitation coil combination. As demonstrated in the preceding section, the multi-coil structure can be regarded as a substitute for a long straight solenoid. Thus, the internal axial direction exhibits a uniform magnetic field. The greater the overall width of the multi-coil structure, the greater the length of the uniform magnetic field region (target area). The magnetic field strength rapidly increased when approaching the multi-coil structure. Once inside, the field strength gradually increased within a certain distance influenced by the coil boundary before stabilizing at its maximum value. Away from the multi-coil structure, the magnetic field strength undergoes a law of change, which becomes axisymmetric when approaches the coil structure. The magnetic field strength outside the coil increased at a greater rate as the overall width of the multi-coil structure increased. On the other hand, the increase inside the coil was slower with longer distance.

The change pattern of the magnetic field gradient was centrosymmetric, with the central symmetry point located in the axial center of the excitation coil combination. The unilateral magnetic field gradient displayed a slow increase followed by a rapid decrease, as it was reduced to zero to maintain a certain length. Based on the change rule of magnetic field strength, on the outside of the multi-coil structure, the magnetic field gradient grows slowly with decreasing distance to the coil structure. Reaching the boundary of the multi-coil structure, the magnetic field gradient reaches its maximum value. Entering the interior of the multi-coil structure, the magnetic field gradient first decreases rapidly to zero and then remains essentially unchanged up to the point of axial center symmetry of the magnetic field gradient. The magnetic field gradient increases faster and decreases slower as the overall width of the multilayer coil structure increases, resulting in a larger maximum value. Therefore, increasing the overall width of the multi-coil structure provides multiple benefits: (i) it expands the delivery range of the magnetic drug and increases its residence time in the magnetic field; (ii) it rapidly increases the delivery speed but slows down its acceleration near the target area, making it easier for it to remain in the target area; and (iii) it increases the aggregation area of the magnetic drug, allowing larger tumors to be treated with a single application, which is extremely beneficial for magnetic targeting therapy. Furthermore, as the overall width of the multi-coil structure increases, the effective range of the magnetic field also extends. This leads to the magnetic drug moving towards the target area over a greater distance, resulting in lower drug concentrations in remote normal tissues and higher concentrations in the target area. As a result, the therapeutic effect is enhanced, and toxic side effects on the organism are effectively reduced.

The effect of the width of a single coil on the magnetic field is described in Section 3.2.1.1. The center of the target area was located at the axial center of the excitation coil combination, as shown by the simulation results. For this reason, the target point is commonly set at the axial center of the excitation coil combination. The target area size is determined by the overall width of the excitation coil.

The coil width should be designed according to the treatment modality and the size of the tumor. For instance, in the case of perfusion therapy for bladder cancer, magnetic drug colloids are perfused into the bladder; hence, it is crucial that the magnetic field effectively covers the bladder in its entirety. Similarly, when administering magnetic drugs intravenously near the tumor area, it is preferable that the magnetic field effectively covers the entire range from the point of injection to the tumor area. For large tumors, excitation coil combination with a larger overall width may increase the size of the target area. For small tumors, the size of the target zone produced can be matched to the tumor’s size by reducing the width of individual coils in the multi-coil structure.

Therefore, the width of the individual coils should match the size of the tumor area. For small tumors, it can be designed to match the size of the target area. This is combined with coil step-by-step shutdown to achieve fast and precise focusing of small focus sizes (see Section 3.2.3 for more information). For large tumors, the overall target area size of the multiple continuously excited coils combination or multi-coil structure may be designed to correspond to the tumor area. The design of the overall width of the multi-coil structure should take into account the treatment modality of the cancer and the required magnetic field strength and gradient. It is important that the magnetic field effectively covers the treatment area while ensuring that the magnetic field strength and gradient meet requirements for magnetic drug targeting. If necessary, the number of coils can be increased to meet the requirements for magnetic field parameters.

#### 3.2.2 Key parameters of delivery coils

##### 3.2.2.1 Inner diameter

The inner diameter of the delivery coil is the inner hole size. The inner diameter of the multi-coil structure was designed to facilitate operation and observation during magnetic targeting therapy. The inner diameter should be large enough to allow for smooth movement of the object to be examined into the inner hole of the multi-coil structure without damaging the coils. The inner diameter should not be excessively large, as this would increase coil resistance, magnetic field strength, and gradient design difficulty, as shown in Figure 3F. The higher the inner diameter of the coil, the longer the wire required to achieve the same number of turns. Consequently, the wire resistance increases, leading to more severe heat generation. When the same current is applied, more energy is required, leading to a higher power requirement for the power supply. However, under the condition that the radial width, width, and number of ampere-turns of the coil remain unchanged, the magnetic field strength and gradient decrease as the inner diameter increases (for this scale range). Furthermore, the internal radial magnetic field difference gradually increased as the inner diameter increased, as shown in Figure 3G. When objects to be examined were placed in different radial positions of the inner hole, the magnetic field parameters varied, resulting in differences in their magnetic targeting therapeutic effects.

Moreover, the magnetic field gradient within the inner hole was significantly smaller than the magnetic field decay rate of the permanent magnet, and there was still a certain level of magnetic field strength in the center of the circle. Therefore, this configuration is well-suited for precise drug delivery to deep tissues in the body. The radial magnetic field of the inner hole had its maximum at the outer boundary and minimum at the center of the circle, showing a nonlinear decreasing law and symmetrical distribution along the radial direction. The gradient magnetic field in the radial direction causes directional movement of the SPIONs in that plane. This facilitates the delivery of the magnetic drug from the inside of the container (blood vessels, bladder, etc., which are tissues or organs used to hold/contain the magnetic drug) to its surface, so that the magnetic drug adheres to the inner surface and thus better interacts with the surrounding cancer cells. In addition, delivery of the magnetic drug in the radial plane of the coil can be achieved by adjusting the relative position of the object to be examined in the radial plane of the inner hole so that the target point is closest to the inner wall of the coil (outer boundary of the inner hole).

Therefore, the inner diameter of the coil should be considered comprehensively to ensure that the object to be examined can enter the inner hole smoothly and does not hinder operation or observation during magnetic drug delivery. This will also ensure therapeutic effects against deep tumors and the enhance the delivery effect of the magnetic drug in the radial plane of the coil.

##### 3.2.2.2 Radial height

The radial height, which refers to the height in the direction of the coil radius, has a direct impact on both the magnetic field strength and the gradient. As the radial width of the coil increases, the number of turns also increases. In the case of applying a given excitation current to a single-turn excitation line, as the radial height increases, the number of turns increases, and the magnetic field strength of the inner hole first increases rapidly and then increases slightly, or even remains almost unchanged, as shown in Figure 3H. When the radial height is small, increasing it results in an increase in the number of turns of the coil or the diameter of the wire, which in turn increases the number of ampere-turns and the magnetic field strength. However, if the radial height exceeds a certain threshold, the magnetic field enhancement of the inner hole is weakly influenced by the magnetic field generated by the outer excitation line owing to its distance from the inner hole. Therefore, the magnetic field strength only slightly increases with radial height or remains essentially unchanged. Further increasing the radial height of the coil at this point will only result in increased coil resistance and heat generation without making any significant contribution to the magnetic field. In the simulation results shown in Figure 3H, the inflection points of the radial height on the magnetic field influence curves varied slightly depending on the width and inner diameter of the coils. The inflection point of the radial height–magnetic field curve gradually shifted as the coil width increased or the inner diameter decreased. For objects of the size of animal or human organs, the inflection point of the magnetic field is typically located within the range of 200–300 mm. Given considerations of magnetic field parameters, energy consumption, and heat generation, the radial height typically does not exceed 200 mm.

#### 3.2.3 Delivery process and current supply

When selecting the target position for the delivery coil structure, it is important to consider the relative positions of the treatment modality and the tumor comprehensively. This will ensure that the target position of the coil corresponds better to the tumor region. The object to be examined is positioned within the inner hole of the delivery coil structure. Target alignment should be performed to align the axial position of the object to be examined, generally the center of the tumor area, with the axial center of the last excited coil combination, which may be a single coil or consecutive multiple coils. This is achieved by using excitation coils to reduce the size of the target area in a step-by-step shutdown manner. The magnetic drug in the axial direction of the coil is moved toward the target area. The position of the target area of the object to be examined in the radial plane of the coil should be adjusted so that it is as close as possible to the outer boundary of the inner hole (inner wall of the coil), and the remaining parts should be kept as far from the outer boundary of the inner hole as possible and close to the center of the coil. In this way, the magnetic drug distributed in the radial plane of the coil moves and aggregates towards the target area. By adjusting the axial and radial positions of the target area of the object to be examined within the inner hole of the coil, two-dimensional targeting motion and target size can be realized.

The excitation method of the multi-coil structure is designed to achieve rapid and precise localized targeting aggregation of magnetic drugs based on the relative position of the target area with respect to the delivery coil structure. With the magnetic targeting coil structure fully excited, the magnetic drug rapidly aggregates near the tumor area. Then, in conjunction with the relative position of the target area, the coil currents are turned off one by one from a point far away from the target area until finally only the coil at the target area is excited. This is achieved by either turning off simultaneously on both sides or turning off coils on both sides one by one (from outside to inside, from left to right, or from right to left), also known as the coil step-by-step turn-off method. This method rapidly reduces the size of the target area of the multi-coil structure. With all coils excited, the magnetic drug is first aggregated within the coil target area as quickly as possible to achieve a rapid reduction in the magnetic drug distribution range. Then, the number of excitation coils is gradually reduced, weakening the magnetic field and decreasing the size of the target area. The movement rate of the magnetic drug is also reduced, allowing the drug to accurately gather and remain in the final target area. The large size of the magnetic drug aggregation area and the deviation between the magnetic drug aggregation area and the target area due to the inertia caused by the fast speed are effectively avoided. The delivery magnetic field provides the best magnetic field parameters at every moving stage of the magnetic drug delivery process to ensure delivery efficiency. Therefore, using the coil step-by-step turn-off method for magnetic drug delivery meets the requirement of small target size and ensures efficient overall delivery.

A set of design methods for the delivery coil structure were formed by analyzing and summarizing the influence of coil parameters on magnetic field parameters. These methods offer guidance for designing *in vitro* magnetic field structures for magnetic drug targeting therapy.

#### 3.2.4 Preclinical delivery system

The study of the magnetic field of the multi-coil structure was combined with the delivery magnetic field parameters determined in the delivery test experiments to develop and manufacture a delivery coil structure used in preclinical magnetic drug targeting therapy for bladder cancer in small animals. Based on the delivery test experiments, it was evident that the gradient magnetic field produced by the delivery coil structure should be equal to or greater than 270 mT, 3.2 T/m. The structure of the delivery coil was designed based on this criterion.

**FIGURE 4:**
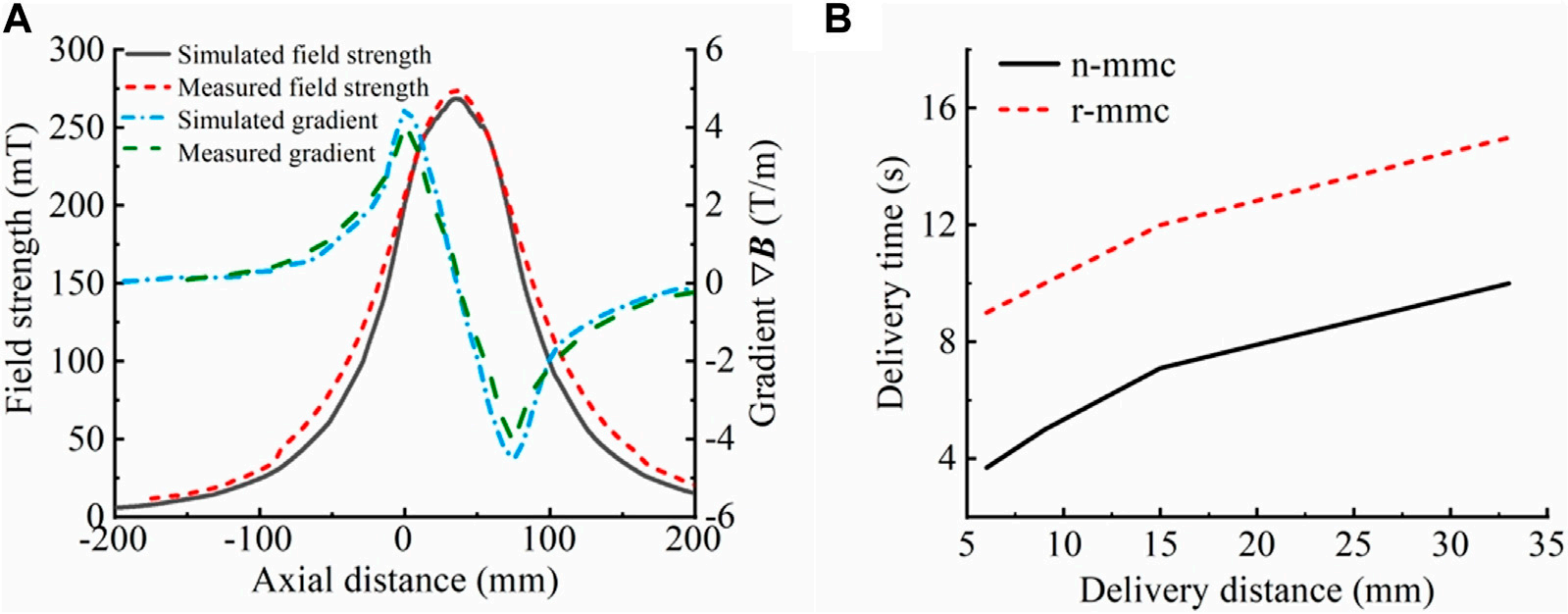
Results of testing of the delivery coil. **(A)** Comparison of simulated and measured magnetic field strength and gradient (simulation results: 269 mT, 4.5 T/m; experimental results: 275 mT, 4.1 T/m). The magnetic field strength differed by 6 mT, whereas the magnetic field gradient differed by 0.4 T/m. **(B)** Delivery times of magnetic drug (n-mmc, r-mmc) saline solution at different distances from the target point. When the distance to the target point was a few millimeters, the delivery took only a few seconds.

Based on the analysis of the coil structure in Section 3.2.1.1, it is recommended that the delivery coil structure be constructed using a hollow multi-coil coaxial close row. The delivery coil structure was designed with an inner diameter of 50 mm to facilitate operation and observation of the rabbit bladder during the *in vitro* magnetic targeting therapy experiment. After considering the size of the rabbit bladder and bladder cancer, the treatment modality (*in vitro* perfusion), we set the spacing between neighboring coils to 5 mm (coil skeleton width), the width of individual coils to 15 mm (to match the size of the bladder cancer), and the number of coils to 3 (to cover the entire bladder). Based on the aforementioned parameters, simulations were conducted to confirm the coil radial height and excitation current. Considering the power supply and coil resistance, the radial height was set to 85 mm, and the number of ampere-turns was set to 8120 A·N. The simulation results are shown in Figure 4A, and the maximum magnetic field strength and gradient were 269 mT and 4.5 T/m, respectively. Three coils with the same structural parameters were manufactured using enameled wire with a diameter of 1 mm, based on the simulation parameters, photographs of the object are shown in Figure 9A. They were made to be coaxially tightly aligned, and a 5 A direct current was applied for excitation to ensure that the magnetic field generated by each coil was in the same direction when excited. The internal magnetic field of the multi-coil structure was measured with a gaussmeter. The maximum field strength and gradient were 275 mT and 4.1 T/m, respectively, and the results are shown in Figure 4A. Both the simulation and experimental results showed that the delivery magnetic field parameters were satisfied. However, there were differences between the simulation and experimental owing to factors such as coil heating, uneven wire distribution, gaps between wires, and inconsistent coil turn numbers. The delivery results of two magnetic drug (n-mmc and r-mmc) salt solutions placed at different axial positions in the inner hole of this delivery coil structure are shown in Figure 4B. Compared with Figure 2, the delivery efficiency of magnetic drugs is higher than that of SPIONs. This is because the overall particle size of magnetic drugs is larger, and agglomeration is more likely to occur under the action of external magnetic fields. Agglomeration increases the overall level of magnetic core, it also increases the magnetic force, thus improving the delivery efficiency. The delivery time was only seconds when the distance from the target was in the millimeter scale.

**FIGURE 5:**
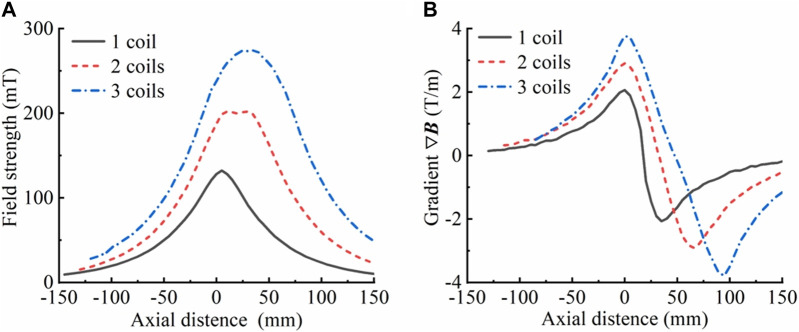
Magnetic field strength and gradient of the actual manufactured coil. **(A)** Magnetic field strength corresponding to different numbers of coils (overall width of the coil). **(B)** Magnetic field gradient corresponding to different numbers of coils (overall width of the coil), where *R* = 85 mm, *r* = 25 mm, *h* = 15 mm, *d* = 5 mm, and a single-turn excitation line passes 5 A current. The axial magnetic field strength was measured with the bottom surface of the lowermost coil as the origin.

To verify the accuracy of the law regarding the influence of coil parameters on magnetic field parameters, magnetic field strength measurements were conducted on one, two, and three coils that had been fabricated by winding. The magnetic field gradients were then calculated. The results, which are presented in Figure 5, indicate that the rule of variation of the magnetic field strength and gradient with the overall width of the delivery coil structure (the number of coils) derived from the simulation is consistent with reality. Therefore, it can be presumed that the simulation results have a certain degree of credibility.

Magnetic fields are of significant interest to the biomedical community owing to their impact on neural stimulation (Panagiotopoulos et al., 2015), magnetohydrodynamic effects, and other biosensing effects in humans and animals (Malkin and de Jongh Curry, 2003). The potential impact of applied magnetic fields on human safety remains uncertain, as their biological effects and specific mechanisms have yet to be fully understood and require further study. However, based on the numerous studies conducted so far, a magnetic field strength of 500–800 mT is commonly used in clinical experiments on animals and humans (Gupta and Hung, 1990; Lübbe et al., 1996b; Kubo et al., 2000). Furthermore, according to a survey, MRI equipment is most commonly used clinically with magnetic field strengths of 1.5 T and 3.0 T (Campbell-Washburn et al., 2019; Sarracanie and Salameh, 2020). MPI devices typically have gradient fields of approximately 4 T/m (Billings et al., 2021), whereas preclinical scanners can have magnetic field gradients of up to 7 T/m (Goodwill et al., 2013; Yu et al., 2017a; Yu et al., 2017b). From this, it can be inferred that the magnetic field strength and gradient (275 mT, 4.1 T/m) produced by the coils of the system presented here fall within the safe dose range for magnetic targeting therapy in both humans and animals and can thus be used with confidence.

## 4 Drug delivery and MPI experiments

### 4.1 Bioavailability of magnetic drugs

**FIGURE 6:**
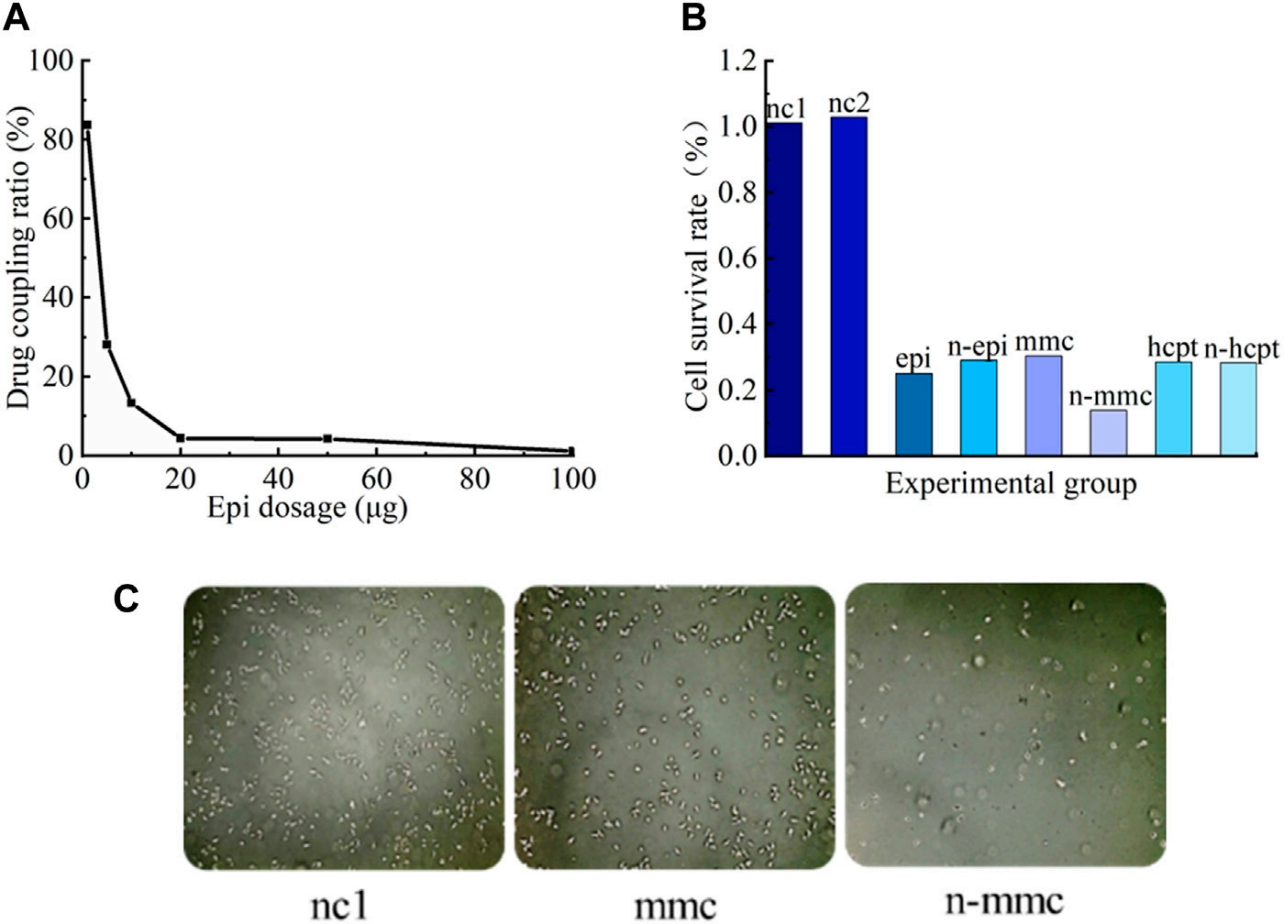
Coupling effects and bioavailability of magnetic drugs. **(A)** Drug-coupling rates of Nanoeast and epi. For each 100 μg of Nanoeast SPIONs, 100 μg, 50 μg, 20 μg, 10 μg, 5 μg, and 1 μg of epi were added for drug coupling to form a magnetic drug, and the absorbance was measured with an enzyme marker to reflect the drug-coupling rate. **(B)** Cell survival rates for different SPIONs, anti-tumor drugs, and magnetic drugs. The addition of the two chosen SPIONs resulted in slightly higher cell survival rates compared with the blank group in its normal culture state. Compared with anti-tumor drugs alone, the magnetic drug had the same killing effect on cancer cells. **(C)** Cell microscopy results of CCK-8 assay for SPIONs (uncoupled Nanoeast), anti-tumor drug (mmc), and magnetic drug (anti-tumor drug-coupled Nanoeast n-mmc). Both magnetic drugs (n-mmc) and anti-tumor agents were substantially effective in killing bladder cancer cells when acting alone (mmc).

The drug-coupling rate of Nanoeast with epi was determined using an enzyme labeling apparatus as depicted in Figure 6A. Epi effectively coupled with Nanoeast to form a magnetic drug, but the coupling rate rapidly decreased with increasing epi drug content. It should be noted that 100 μg of magnetic spheres cannot be fully loaded with 1 μg of epi.

The results of the bioavailability validation experiment for magnetic drugs are shown in Figure 6B. When unmodified Nanoeast and Resovist were injected on T24 cancer cells, the survival rates of the cells were marginally higher than those of the cells in the control group (injected with nothing). The survival rate of cancer cells in the nc1 group (injected with unmodified Nanoeast) was 101%, whereas that in the nc2 group (injected with unmodified Resovist) was 103%. These results show that neither of the two selected superparamagnetic nanoparticles exhibited significant cytotoxicity. Resovist and Nanoeast consist of γ-Fe_2_O_3_ (maghemite) and Fe_3_O_4_ (magnetite), respectively. The biocompatibility of ferromagnetic oxides has been extensively demonstrated (Weissleder et al., 1989; Muldoon et al., 2005); some MNPs have received registration approval for direct application to the human body, and others are currently undergoing clinical trials (Assa et al., 2017). Therefore, choosing safe and dependable MNPs for use as drug carriers and for targeted transport is not difficult. Furthermore, the cell survival rates in both the nc1 and nc2 groups exceeded 100%, indicating that the treatments promote cell growth to some degree, although their mechanism of action remains unclear and their generalizability requires further investigation.

The killing effects of chemotherapeutic drugs on cancer cells prior to and after coupling with SPIONs appear to be equivalent; in both cases, they could effectively kill a large number of cancer cells and significantly hinder tumor growth. Therefore, both the selected SPIONs could be used as effective carriers of magnetic drugs. In this scenario, the impact of the magnetic drug combination cannot be assessed solely by the individual effects of magnetic intervention or drug intervention, which involves the mechanism of drug action. The effect of magnetic drug n-mmc, as shown in Figure 6B, was inconsistent with those seen in other groups, possibly owing to the inconsistency between the intracellular and extracellular action effects of mmc. Further research is necessary to address this specific concern.

The CCK-8 microscopic results, obtained via large-scale observations using statistical principles, are shown in Figure 6C, where the bright white spots represent the surviving cancer cells. Both magnetic drugs (n-epi, n-mmc, n-hcpt) and chemotherapeutic drugs (epi, mmc, hcpt) acting alone could kill bladder cancer cells substantially and effectively.

### 4.2 *In vivo* imaging capabilities of the MPI system

MPI can be used to perform point-by-point detection imaging of the entire detection range by adjusting the position of the field-free-point (FFP) supplied by the selection field. Oscillatory changes and magnetization changes of SPIONs at the FFP are induced by the alternating magnetic field of the driving field. The magnetic signals generated by the SPIONs are received by the detection coil, and the SPIONs in the detection area are localized and quantitatively imaged (Gleich and Weizenecker, 2005; Rahmer et al., 2009; Goodwill and Conolly, 2010; Goodwill et al., 2012). The precise localization and detection of SPIONs can be achieved by the movement of the selective field, as the strong gradient magnetic field of the selective field saturates magnetization of all SPIONs in the region except for FFP and suppresses the driving field (Harvell-Smith and Thanh, 2022). At the FFP, the higher the content of SPIONs, the larger the magnetic signal that is produced, so SPIONs can be quantitatively imaged (Pablico-Lansigan et al., 2013; Saritas et al., 2013). When a sufficient amount of SPIONs is present in the detection area to alter the signal of the detection coil, the corresponding position on the MPI detection image will display a color change. The darker the color on the MPI detection image, the higher the concentration of SPIONs. For instance, a blue color is indicative of the absence of SPIONs, whereas a weak concentration of SPIONs will result in a yellow-green color, and a high concentration will be depicted by a red color. Thus, the spatial distribution of SPIONs can be ascertained via the size and location of the color-altered area in the MPI image, and the depth of the color can be used to establish the concentration of SPIONs.

**FIGURE 7:**
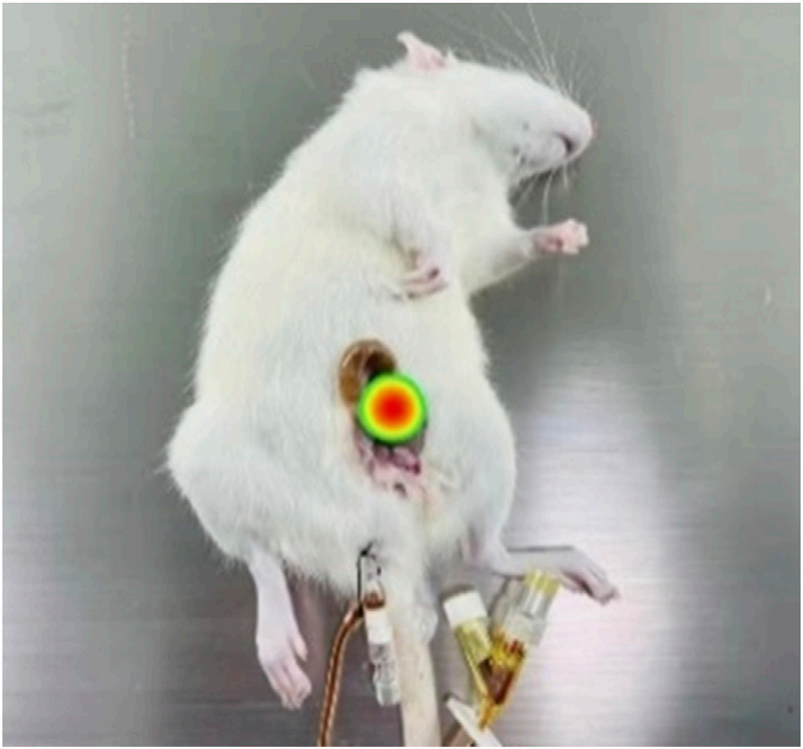
*In vivo* imaging of magnetic drugs in the rat bladder with MPI.

The imaging results for the magnetic drugs within the rat bladder obtained through MPI and the synthesized results of the rat photographs are shown in Figure 7. Based on the MPI detection principle, the imaging location, the size of the red area, and the uniform distribution of the color, it could be seen that the magnetic drug was in the bladder of the rat (red area in the figure) and uniformly distributed throughout the whole bladder, consistent with the reality. Therefore, the findings of the experiment show that the magnetic drugs in animals can be precisely imaged by the MPI equipment.

### 4.3 Anti-tumor drug delivery and imaging experiments in rabbit bladder

The bladder is a hollow organ, and bladder cancer is commonly located on the surface of its mucosal lining (Batista et al., 2020). During treatment, diluted anti-tumor drugs are perfused into the bladder to make direct contact with the cancer cells in the mucosal lining (Ren et al., 2016; Bao et al., 2020), without the need to administer the drugs through vascular injection or other means. Therefore, bladder cancer is suitable for magnetic drug targeting therapy without the formation of thrombus blockages and other problems.

**FIGURE 8:**
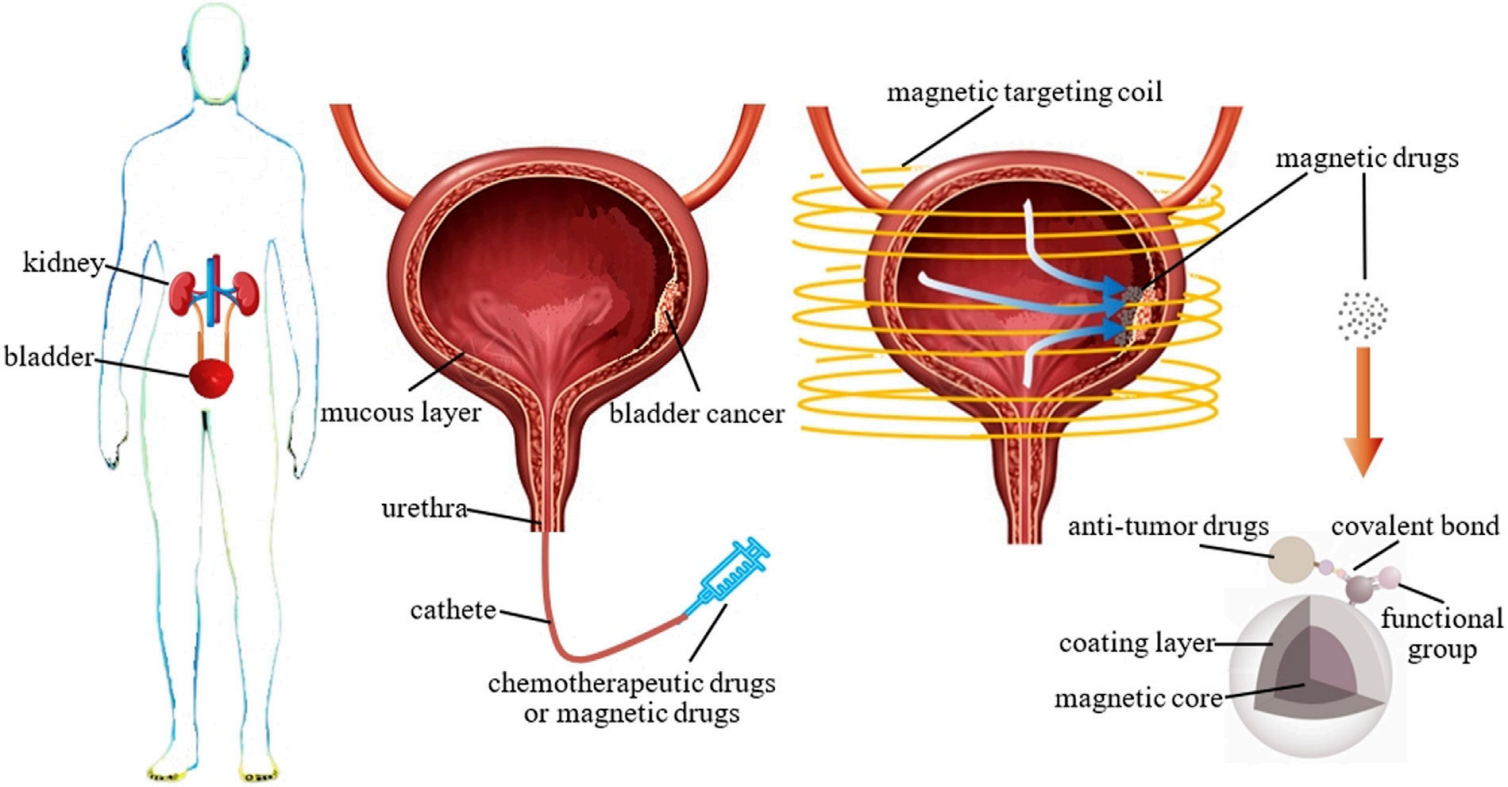
Bladder cancer and its treatment modalities. Left: the location of the bladder in the human body; middle: a bladder cross-section showing the location of bladder cancer and the drug delivery modality of traditional perfusion therapy and magnetic drugs; right: schematic diagram of magnetic targeting therapy for bladder cancer with magnetic drugs and composition of magnetic drugs.

Traditional bladder perfusion therapy involves the use of a catheter to deliver diluted anti-tumor drugs into the bladder. The patient’s posture is adjusted by constantly turning them over so that the anti-tumor drugs come into contact with the cancer cells on the mucosal lining of the bladder (Figure 8, middle). After being retained for a certain period of time, the perfusion can be drained through urination (Raven et al., 2018; Babjuk et al., 2019; Volovat et al., 2020). Bladder perfusion chemotherapy differs from general chemotherapy in that it only affects the mucosal surface of the bladder and does not enter the systemic circulation. However, the entire lining of the bladder is exposed to the anti-tumor agent, causing damage to the normal bladder area. Thus, actual localized treatment is not achieved. In contrast to traditional bladder perfusion therapy, magnetic targeting therapy uses an extracorporeal magnetic field to deliver anti-tumor drugs carried by SPIONs in the perfused magnetic suspension, which aggregate and are retained at tumors (Figure 8, right). This technique significantly lowers the drug concentration in normal mucosal sites and raises it at the tumor site, thereby decreasing the damage to healthy tissues generated by anti-tumor drugs while providing a more effective tumor-killing outcome. This is of great benefit to the patient undergoing cancer treatment and to the entire medical community.

**FIGURE 9:**
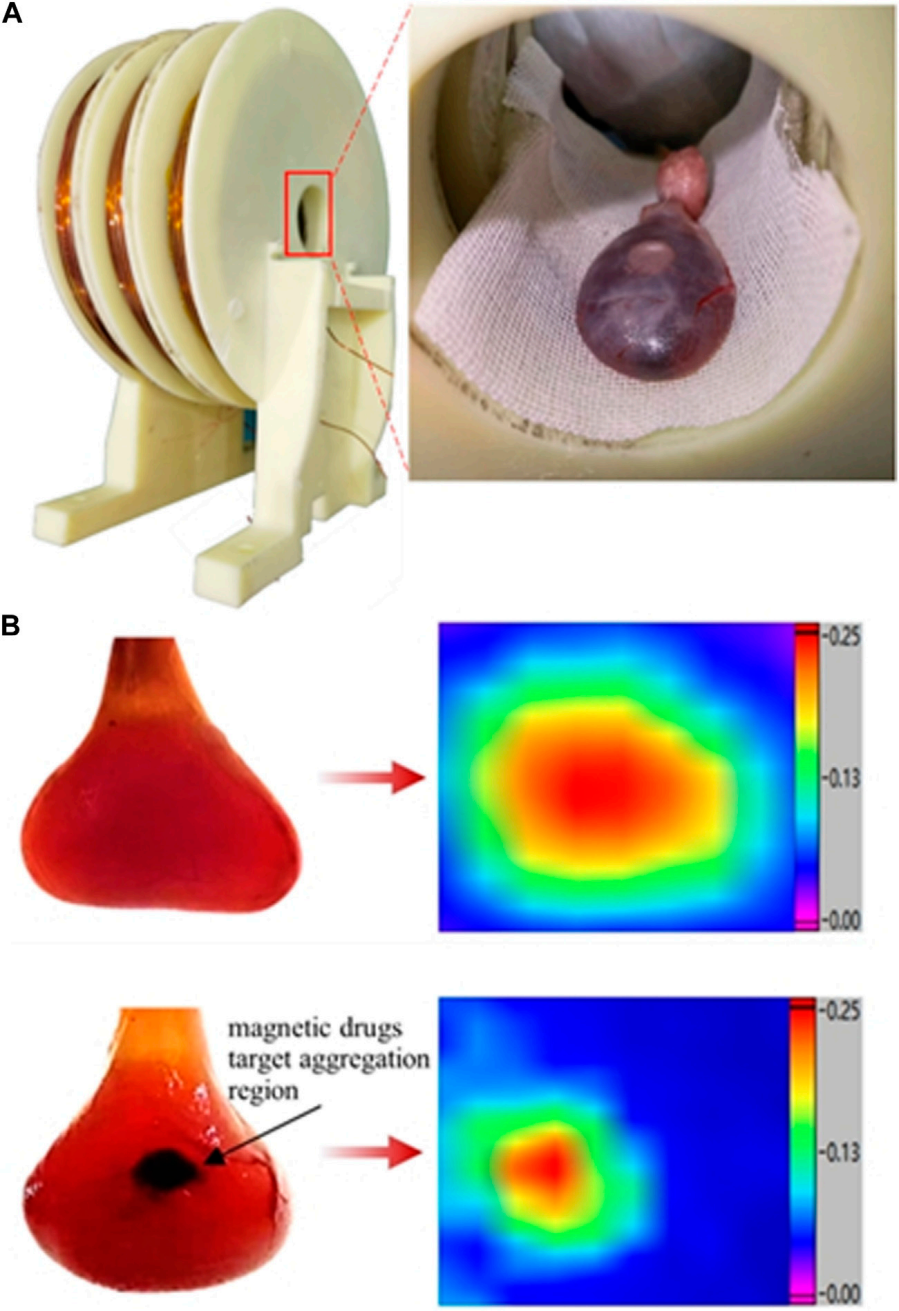
Structure of the delivery coil and status of magnetic drugs in rabbit bladder before and after delivery. **(A)** Delivery coil structure and bladder placement in delivery coil. **(B)** Physical state of rabbit bladder and MPI results before and after delivery. Top: before delivery; bottom: after delivery; left: physical state of the bladder; right: MPI results showing magnetic drugs in the bladder. The physical state of the object was captured with a high-pixel camera using a positive backlighting lighting method (lighting from directly behind the object).

Here, bladder perfusion *in vitro* experiments were performed to validate the magnetic drug delivery capability of the developed system. A 2.5-kg female rabbit was fatally embolized by air gas injection through auricular vein. The bladder was excised and cleansed, followed by the perfusion of 100 uL mmc-modified Nanoeast 30 nm SPIONs mixed with 4 mL saline. The magnetic drug suspension solution in the bladder appeared homogeneous, without any noticeable magnetic drug microclusters. The physical state of the solution in bladder is showed in the top left panel of Figure 9B. The results of its MPI detection are showed in the top right panel of Figure 9B, where the red area is broad and the color distribution is uniform. Based on the MPI imaging principle, the magnetic drug was uniformly distributed throughout the bladder, which was consistent with the situation shown in the top left panel of Figure 9B. It was then placed in the inner hole of the magnetic drug delivery coil structure designed for magnetic targeting therapy, as shown in Figure 9A. The bladder target point in the axial direction of the coil was precisely aligned with the axial center of the multi-coil structure. The relative position of the bladder target point in the radial plane was adjusted so that the bladder target point was attached to the surface of the outer boundary of the inner hole (inner wall of the coil), and the rest of the area was as close as possible to the center of the coil. After immobilizing the rabbit’s bladder, a magnetic drug targeting treatment lasting 12 s was carried out. To achieve rapid and precise targeting therapy, the size of the target area was reduced by turning off the excitation coil step by step. Initially, all three coils were excited to expedite the movement of the magnetic drug, which was uniformly dispersed throughout the bladder, toward the target area, thereby forming the initial aggregation. Then, the left- and right-side coils of the delivery coil structure were turned off, leaving only the middle coil excited. Given the reduced range of the magnetic field and size of the target area, the magnetic drug moved further toward the new target area, forming the final aggregates. The physical state of the bladder after magnetic targeting is shown in the bottom left panel of Figure 9B, where aggregation of the magnetic drug can be clearly observed (black area in the figure). This was effectively demonstrated by the MPI detection results, which showed a significant reduction in the area of the red region in the bottom right panel of Figure 9B. After delivery, the distribution of magnetic drugs was significantly reduced, effectively reducing the damage caused by anti-tumor agents to normal tissues.

The peak values measured in the bottom right panel of Figure 9B were approximately equivalent to those shown in the top right panel of Figure 9A. In general, after delivery, the concentration of SPIONs in the aggregation region increases, and the MPI detection signal should be greater than the homogeneous state before delivery. However, upon delivery, the SPIONs were subjected to magnetic force, aggregated in close proximity, and agglomerated, and the increase in particle size resulted in reduced particle rotation, leading to weakening of the MPI response signal. Therefore, under the dual mechanism of MPI signal enhancement by increasing concentration and MPI signal reduction by particle clustering, the MPI signal after delivery remained essentially the same as before delivery. Whether the MPI signal increases or decreases before and after delivery is affected by the parameters of the delivery magnetic field, the particle size of the SPIONs, the material of the coating layer, the surface charge potential, the MPI magnetic field parameters, and other aspects, and the specific mechanism of the effect needs to be confirmed by further research. However, it is certain that MPI technology can be used to detect and image the aggregation state and location of magnetic drugs after magnetic targeting therapy.

The effectiveness of magnetic drug targeting therapy was demonstrated by comparing actual photographs of the rabbit bladder *in vitro* before and after treatment with the MPI detection results. In addition, the size of the drug aggregation area after magnetic targeting suggests that the developed system can cover a target area of 5 mm × 5 mm.

## 5 Discussion

In this study, the targeted delivery ability of SPIONs, which meet the necessary conditions for MPI imaging, under external magnetic field is analyzed theoretically and verified experimentally. A theoretical model was developed to explain the magnetic targeting motion of SPIONs at the micro-nanometer scale. The mechanism by which magnetic field parameters affect the motion of SPIONs was revealed. The necessary conditions of magnetic drug delivery and the key parameters of drug delivery system were clarified. A design method for the delivery coil structures and principles for the selection of magnetic drug carriers when performing magnetic targeting therapy are proposed. It provides guidance for magnetic field design in different scenarios and different disease delivery treatment.

Experimental studies showed that single-core and multi-core SPIONs exhibit different magnetic parameters under the same delivery conditions. The different delivery effects of the two types of SPION were consistent with the proposed theoretical model of magnetic targeting at the micro-nanometer scale. In addition, the multi-coil structure was designed to produce a precise magnetic field with both high strength and high gradient, facilitating the delivery of SPIONs to specific locations. Using three coaxial coils arranged in close proximity within the parameters described in this paper, a delivery magnetic field with a maximum strength of 275 mT, a gradient of 4.1 T/m, and a target area size of 5 mm × 5 mm was constructed with step-by-step coil turn-off, effectively realizing the rapid and precise delivery of magnetic drugs. This was verified by a rabbit bladder experiment *in vivo*, and the results were observed under MPI.

The theoretical analysis and experimental results both show that the magnetic force is related to the magnetic field strength and gradient when the nanoparticles are unsaturated magnetized, as a result of joint action. After saturation magnetization, the magnetic field force is only related to the magnetic field gradient. In addition, the magnetic field strength enables the nanoparticles to overcome the maximum static friction force $F_{m0}$ and/or cell adhesion forces to begin to move, whereas the magnetic field gradient provides acceleration during the movement. Therefore, when designing a delivery magnetic field, it is enough for the magnetic field strength to meet the requirements; for the magnetic field gradient, the larger, the better. In the case of unsaturated magnetization, by adjusting a single parameter (e.g., increasing the strength or decreasing the gradient), SPIONs can be made to move at the same velocity, achieving the equivalent replacement of the delivered magnetic field parameters. This discovery overcomes previous limitations on the structure and parameters of the delivered magnetic field and makes its structure more varied, leading to the possibility of designing a more flexible delivered magnetic field structure with conveniently controllable parameters.

Moreover, a hollow multi-coil structure with a coaxial close arrangement was utilized to construct the delivery coil structure instead of a conventional electromagnet or permanent magnet structure. The object to be examined is placed in the inner hole, which effectively enhances the magnetic field strength and gradient. This provides a section of uniform magnetic field with maximum field strength in the axial direction of the coils, facilitating the aggregation and retention of magnetic drugs in the axial direction. The magnetic field strength decays slowly in the radial direction of the coil, such that a strong magnetic field strength is retained at the center of the circle, making it suitable for deep tissue detection. Magnetic drugs can be delivered and aggregated in the radial plane owing to the radial gradient magnetic field. Therefore, during magnetic targeting, the target point of the object to be examined should be aligned with the axial target point of the coil. Then, its relative position should be adjusted in the radial plane to be as close as possible to the outer boundary of the inner hole of the coil. The remaining area is kept as close as possible to the center of the coil. By adjusting the axial and radial positions of the target area of the object to be examined within the inner hole of the coil, two-dimensional targeting motion and target point sizing of the magnetic drug can be achieved. By designing the width of the individual coils, the delivery coil structure can be flexibly adapted to different target zone sizes. With the step-by-step coil turn-off model, rapid and precise aggregation of small target size can be effectively realized.

The rabbit bladder anti-tumor drug delivery experiment described in Section 4.3 was performed *in vitro* by removing the rabbit bladder from the body. More research is required to confirm the efficacy of magnetic targeting therapy on the bladder *in vivo*. However, the core of magnetic targeting therapy is the magnetic drug carrier SPIONs, which deliver anti-tumor drugs to the required target under the action of an external magnetic field. The delivery motion is affected by the magnetic field strength and gradient. Compared with *in vivo* experiments, apart from the differences in biological organization, the main challenge for magnetic targeting therapy lies in the strength and gradient of the magnetic field at the target region. Notably, in *vitro* experiments, the bladder is closer to the magnetic field, whereas in *vivo* experiments, it is distant from the magnetic source owing to the effects of biological tissue structure and other factors. However, in traditional magnetic targeting therapy, where permanent magnets are applied *in vitro*, the problem of rapid decay of the magnetic field with increasing distance also exists. In this study, magnetic targeting therapy was performed by placing the object to be examined in the inner hole of the multi-coil structure, allowing the problem to be effectively solved by utilizing the composite gradient magnetic field generated by the multi-coil structure (an axial gradient magnetic field with a section of uniform magnetic field, where the length of the uniform magnetic field can be controlled: the radial magnetic field decays slowly, whereas the magnetic field strength is still strong at the axis of the smallest magnetic field strength in the radial plane of the multi-coil structure).

In order to generate DC magnetic field with high field strength and high gradient, the coil turns are more and the current is larger. From the heat generated by the wire *Q* = *I* ^2^ *R*△*t*, it can be seen that the wire heat *Q* is proportional to the length of the wire *L* (*R* = *ρL*/*S*), the passing current *I* and the excitation time △*t*. Therefore, when working for a long time, the coil heat is more serious. The system designed in this study was used for *in vitro* experiments of small animals without the use of cooling devices. Because of the small size of the organs or tissues of small animals, the time required for the delivery process is short, usually tens or tens of seconds (12 s for the rabbit bladder experiment). As a result, when the equipment is working, the single excitation time is short, and the coil heating is not serious. No effect on organisms or biological tissues. Subsequent devices for *in vivo* animal or human experiments will require larger magnetic fields and will therefore have additional cooling links or be made of superconducting materials instead.

Experimental results showed that anti-tumor drugs could be effectively coupled to SPIONs without any loss of potency. The delivery system was designed to perform magnetic drug targeting enrichment, and the results before and after magnetic drug delivery were observed using MPI. This provides a good experimental basis for the use of MPI to monitor the movement of magnetic drugs and their aggregation state during magnetic targeting therapy.

Multi-core Resovist SPIONs of small size can be delivered by a weak field strength and gradient at a relatively low speed, but single-core Nanoeast SPIONs of larger particle size require a stronger field strength and gradient to be delivered at a much higher movement speed. These results serve as a foundation for magnetic drug carrier selection during magnetic targeting therapy. Multi-core, small-particle-size SPIONs such as Resovist are preferable when the applied delivery magnetic field is weak. However, if the applied delivery magnetic field is strong, single-core, large-particle-size SPIONs such as Nanoeast may be more suitable. When choosing a magnetic drug carrier for magnetic targeting therapy, it is important to consider these factors.

However, it should be acknowledged that the non-specific adsorption and phagocytosis of SPIONs by cells cannot be avoided in clinical applications; these problems need to be addressed in future magnetic targeting experiments.

Further developments in MPI technology are expected to enable observation of the *in vivo* magnetic targeting therapy process, including dynamic display of the trajectory, local concentration, aggregation state, and location of magnetic drugs in real time. Such advances would provide effective technical means and assurances for further research and development of magnetic targeting therapy (determination of the parameters of magnetic field required for magnetic drugs delivery and magnetic targeting coil structure, research into how magnetic drugs enter the cell, etc.). Promotion of this technology is expected to lead to advances in cancer treatment and acceleration of industrial development.

## 6 Conclusion

In this study, a comprehensive investigation of SPION materials, magnetic drug bioavailability, MPI of magnetic drugs *in vivo*, and a magnetic drug delivery system was carried out. The drug delivery and MPI device developed here were validated. Experimental results demonstrated that the SPIONs could effectively be coupled to anti-tumor drugs without compromising their potency, and that the designed drug delivery system can effectively perform magnetic drug targeting enrichment and is suitable for observation of magnetic drug delivery by MPI. This study thus provides a comprehensive theoretical and practical reference to facilitate the combined utilization of magnetic drug delivery and MPI technology.

Currently, the magnetic drug delivery system and MPI system presented here are independent of each other. In future research, the MPI system will be improved and refined to include drug delivery functionality. Preparations are also underway to construct delivery devices with a larger target space (i.e., the size of the inner hole) with superconductors for delivering drugs to humans. The magnetic field strength and gradient of the device are greater, the delivery efficiency is higher, and the cooling link makes the device safer and more reliable to use. The relevant research has been carried out in cooperation with clinical institutions and in accordance with ethical guidelines.

## Data Availability

The original contributions presented in the study are included in the article/supplementary material, further inquiries can be directed to the corresponding authors.
